# “I Think You Covered the Three Levels of Drugs and Consent”: Qualitatively Testing Different Operationalizations of an Alcohol and Other Drugs-Involved Sexual Violence

**DOI:** 10.1007/s10508-024-02947-w

**Published:** 2024-07-22

**Authors:** Nili Gesser, Benjamin W. Katz, Tiffany Chiu, Ellei M. Burmeister, RaeAnn E. Anderson

**Affiliations:** 1 Department of Criminology and Justice Studies, Drexel University, 3401 Market Street, Philadelphia, PA 19122, USA; 2 Department of Psychology, University of Wisconsin-Milwaukee, Milwaukee, WI, USA; 3 Health & Behavior Studies, Department of Education, University of North Dakota, Grand Forks, ND, USA; 4 Department of Psychology, University of North Dakota, Grand Forks, ND, USA; 5 Alluma, Crookston, MN, USA

**Keywords:** Alcohol and other drugs (AOD), Sexual violence, Cognitive interview, Consent, Rape, Sexual Experiences Survey

## Abstract

Substance-involved rape is increasing among college students, particularly women (Koss et al., 2022). Addressing rape requires first measuring it accurately in surveys to understand its true scope and nature. We used cognitive interviews with 40 young adults to qualitatively test the construct validity of an alcohol- and other drugs (AOD)-involved rape item in the Sexual Experiences Survey by asking participants to comment on different operationalizations of this construct. Our findings revealed that different phrasings elicited different interpretations of the items by participants. Specifically, the results indicated that (1) respondents viewed the different operationalizations as a sequence of events with varying severity; (2) some participants focused on the intentionality and responsibility of the perpetrator as opposed to opportunistic perpetration; and (3) study participants consistently chose one of the operationalizations as describing “being roofied” (being drugged without consent). Participants also contributed additional scenarios not described in the questionnaire and shared their interpretations of the items. The results underscore the importance of refining survey language to properly measure AOD-involved rape and allow us to understand how to tailor appropriate questions for best comprehension. The findings indicate the benefit in including several items about AOD-involved rape in questionnaires such as the Sexual Experiences Survey, with each item addressing different scenarios of victim intoxication. The results could also have important implications for sexual violence prevention programs, which should discuss consent, intentions, and responsibility specifically in the context of AOD consumption.

## Introduction

Sexual victimization, defined as sexual activity that occurs without consent, affects many American women. Rape, the most extreme form of sexual victimization, is non-consensual, penetrative sex via substance incapacitation, threats of force, or physical force (Basile et al., 2014). Approximately 20% of college women experience rape on campus (Muehlenhard et al., 2016), and at least 80% of rapes that occur during the college years are alcohol-involved (Koss et al., 2022). The centrality of alcohol consumption in the occurrence of rape has been steady for decades (Koss et al., 2022). Rape in and of itself is often damaging, resulting in a range of negative mental health consequences, including depression, PTSD, and substance use problems (Dworkin et al., 2017). Those who experience alcohol-involved rape rather than non-impaired rape often experience additional negative outcomes, greater feelings of self-blame (Littleton et al., 2009) and more negative reactions from social supports (Peter-Hagene & Ullman, 2015).

Yet, studying alcohol-involved sexual violence is methodologically complicated to measure due to the pharmacological, psychological, and social effects of alcohol (Lorenz & Ullman, 2016). For example, difficulties in the assessment of alcohol-involved sexual violence can be relatively challenging due to demographic differences in rates of alcohol metabolization (Thomasson, 2002), which may systematically contribute to differences in impairment and ability to recall incidents of alcohol-involved victimization. Alcohol has pharmacological effects on cognitive functioning (Steele & Joseph, 1990) and can contribute to a misperception of sexual consent-related cues (Jozkowski et al., 2014; Muehlenhard et al., 2016), which may further contribute to inaccurate recall of incidents of alcohol-involved sexual victimization. Moreover, different social scripts and expectancies surrounding the use of substances during sexual encounters may result in misperception of sexual consent and subsequent (under) reporting of AOD-involved sexual victimization.

Among the different types of tactics involved in sexual violence, such as verbal coercion and physical force, substance-related tactics tend to have the lowest reliability ratings for women (Anderson et al., 2021a). This may be due to the myriad ways substances can be involved in an incidents of sexual victimization: AOD could have been consumed voluntarily or involuntarily (including unknown and coerced or pressured consumption); AOD can affect consent and resistance; and AOD can be used as an overt or covert tactic of perpetration. For example, perpetrators may use tactics such as placing date rape drugs or illicit substances (e.g., Rohypnol or “roofies”) into one’s drink (Lahane & Kaur, 2022), which is a tactic used in drug- and alcohol-facilitated sexual victimization (Crawford et al., 2008).^1^

Additionally, some measurement challenges are due to the phrasing of instruments in surveys. In their study, Palmer and Perrotti (2016) found that different wording on two sexual victimization surveys that were administered to college students at the same university resulted in vastly different estimates. Discrepancies were especially noticeable for sexual victimization due to incapacitation. They concluded that, “estimates vary greatly depending on the survey methodology and question wording” (Palmer & Perrotti, 2016, p. 413).

The goal of this paper was to examine the construct validity of three different survey items measuring AOD-involved sexual violence using a cognitive interviewing paradigm to determine how participants perceive and interpret these different items. We consider AOD’s role both in victimization, or being harmed by sexual violence, and in perpetration, or instigating incidents of sexual violence.

### The Measurement of Alcohol and Other Drugs-Involved Sexual Violence

Despite decades of evidence linking alcohol and sexual violence, AOD-related interventions and sexual violence interventions have been largely siloed (Leone et al., 2022). One barrier to more efficacious research on AOD-involved sexual violence is measurement. Operationalizing and studying AOD-involved sexual violence are challenging for multiple reasons. AOD can be used as an explicit tactic to coerce—pressuring someone to drink or giving them a secretly spiked drink. These scenarios reflect involuntary consumption by the victimized. Voluntary consumption can also be weaponized—even if not incapacitated, AOD impair a person’s ability to resist, decrease inhibition, and result in incapacitation and even loss of consciousness when consumed in excess (Pumphrey-Gordon & Gross, 2007). These varying effects pose significant challenges in discerning the threshold at which an individual is too intoxicated to communicate sexual consent (Leone et al., 2022).

This diversity has resulted in numerous methodological approaches researchers have employed to study the complex interplay between alcohol consumption and sexual violence. The Sexual Experiences Survey (SES) is the most commonly used measure to determine whether an individual has experienced sexual violence; it can be used to measure victimization or perpetration, depending on which form is used (Anderson et al., 2021b; Fedina et al., 2018). However, a systematic review recently found that the SES is modified most of the time it is administered, most often by adding alcohol and substance use-related items (Anderson et al., 2021b). Additionally, in a mixed-method study that tested gender differences in sexual violence perpetration behaviors and the validity of the reports of these behaviors, Jeffrey and Senn (2024) have found that some participants (both male and female) struggled with understanding the term “to take advantage of someone” in the context of alcohol consumption; the researchers pointed to the need to update the language in the SES (perpetration) and other measures of sexual violence. Palmer and Perrotti (2016) also found that estimates of sexual violence, in particular those related to incapacitation, are influenced by the phrasing of survey items. These findings suggest there is a researcher-based demand for more effective ways to operationalize AOD-involved sexual violence.

Research further indicates that the SES may measure AOD-involved incidents with differing degrees of reliability between genders, with alcohol-related tactics being more accurately reported by men than by women (Anderson et al., 2021a). These discrepancies could partially stem from biological differences in alcohol metabolization, as men generally metabolize alcohol more efficiently than women (Thomasson, 2002). Consequently, women might experience greater and faster impairment from alcohol, potentially impacting their ability to accurately recall alcohol-involved incidents of victimization.

In addition to quantitative research highlighting potential challenges in reliability, qualitative research has also underscored how commonly used terms are interpreted in variable ways. Ullman et al. (2019) found that adult women who were sexual assault survivors from the Chicago, Illinois area did not reliably distinguish terms like “impaired” (some impact of alcohol on decision-making) from “incapacitated” (strong impact of alcohol to the point of being unable to behave, think, or decide normally). This finding could hinder accurate measurement of sexual violence incidents, since researchers may use the terms in surveys to indicate distinct states and different levels of the impact of alcohol. Yule and Grych (2022), using cognitive interviewing, demonstrated how even when participants were instructed to exclude acts that might represent joking around, they still included these behaviors in their responses. This suggests that instructions used in questionnaires like “do not consider times you were incapacitated” to differentiate impaired and incapacitated alcohol-involved sexual victimization may not be useful (e.g., in the new SES-V; Koss et al., 2024). This also suggests the need for further qualitative work to inform measurement research, so that researchers can find and develop measurements of AOD-involved sexual violence that use terms in a way participants and researchers understand similarly.

Another challenge in operationalizing alcohol-involved sexual violence is the consideration of other substances. AOD are often consumed simultaneously (Gillman et al., 2018; Tzilos et al., 2014); thus, including questionnaire items that only query about alcohol or only query drugs may result in underreporting as participants who co-use may think a single substance item does not necessarily reflect their experience. Further, there may be synergistic effects from certain combinations of alcohol and drugs that may facilitate different forms of sexual violence. For example, ketamine is a very fast acting anesthetic that could cause immediate trance-like symptoms and hallucinations which facilitating violence. While cannabis is the most common drug used and is often associated with alcohol-involved assaults, the mere range of possible drugs that could be associated with sexual violence, voluntarily (Hagemann et al., 2013) or involuntarily, can present a challenge to research (Costa et al., 2020; Fiorentin & Logan, 2019).

All these complexities underscore the need for nuanced approaches to operationalizing and studying the diverse ways AOD can be utilized as a tactic of sexual violence, particularly considering the distinct experiences and vulnerabilities faced by survivors in these contexts. Much of the prior research investigating optimal measurement strategies and highlighting mechanisms of measurement discrepancy has focused on questionnaire features like response formats (Anderson & Cuccolo, 2022) or global issues like content (Anderson & Delahanty, 2020; Strang et al., 2013. Relatively fewer articles focus on measurement related to specific tactics, including AOD-involved sexual violence. A focus on alcohol specifically may be especially beneficial given the variety of ways alcohol can be involved in sexual violence and how alcohol affects sexuality (Frank et al., 2020; Jeffrey & Senn, 2024; Johnson et al., 2022). Indeed, the revised SES-V which debuted in 2023 includes five times as many alcohol-related tactics as the previous 2007 revision (Koss et al., 2007, 2024). Yet, none of the 2023 items nor the 2007 items have been subject to cognitive interview type validity testing to ensure that participants understand the items in the way researchers intend (Willis, 2004).

### Cognitive Interviews

Cognitive interviewing is a qualitative technique intended to improve survey design by ensuring respondents interpret items on the questionnaire the way they were intended to be interpreted (Beatty & Willis, 2007). In this method, people are asked to “think aloud” while they respond to questionnaire items, usually with verbal probes to further understand their reasoning in selecting the response they chose (Luckett et al., 2022). Cognitive interviewing has been used in survey development, refinement, and adaptation to improve respondents’ interpretation of survey items (Ryan et al., 2012).

Cognitive interviews thus offer a multifaceted approach to improving survey and questionnaire design. By delving into how respondents interpret and process questions, these interviews pinpoint potential issues with wording, comprehension, and response processes, facilitating refinement before full-scale implementation (Willis & Artino, 2013). Moreover, they bolster the validity and reliability of research data by uncovering sources of response error or misunderstanding, enhancing the overall quality and trustworthiness of findings (Ryan et al., 2012). Additionally, through techniques like think-aloud and verbal probing, cognitive interviews yield qualitative insights into respondents’ thought processes, providing valuable contextual information to complement quantitative survey results (Luckett et al., 2022). Furthermore, these interviews promote participant-centered design, fostering inclusivity and responsiveness in study approaches, thereby enhancing participant engagement and trust in the research process (Burkhalter et al., 2018).

Cognitive interviewing in combination with thematic analysis has been previously used in sex trade and violence research to improve survey methodology in various ways (Gerassi et al., 2023; Yule & Grych, 2022). For example, in Gerassi et al.’s study, participants’ responses indicated that measuring sex trade involvement with a single item that only addresses “financial compensation” is not comprehensive enough and does not capture instances of sex trade involvement due to exploitation or empowerment. Additionally, respondents endorsed various terms to replace “sex trade” such as “being paid to be sexual” and recommended using a multi-item measure with these different terms.

### The Current Study

The goal of the current study was to examine the construct validity of three different SES-based items that examine alcohol as a tactic of sexual victimization. In spite of the SES being in use for decades, no prior research has conducted cognitive interviewing with any of the items used in this study to understand whether participants understand them as intended. This is particularly important given the complexities and nuances of AOD-involved sexual violence. We chose to use tactic-first items given recent research supporting the strong validity of these items (Abbey et al., 2021; Anderson et al., 2021a). We chose three items that represented a range of how alcohol can be used as a tactic, spanning voluntary vs. involuntary consumption and impairment vs. incapacitation (see Table 1). Items A and C were taken directly from Abbey et al. (2005). Item B was adapted from the SES-Long Form (Koss et al., 2007). We chose a cognitive interviewing paradigm (Willis, 2004) to interrogate the construct validity of these items so that participants’ potential differential interpretation and processing of these items could be captured and used to improve the measurement of alcohol-involved sexual violence. Our findings highlight thematic content that arose directly in response to our planned prompts as well as other themes that arose unexpectedly.

## Method

### Participants

Since there are no established guidelines on the right sample size for cognitive interviews (Luckett et al., 2022), our target sample size was 40 participants. Participants were college-aged students at a public university in the Upper Midwest and community members recruited nationally in the United States. Respondents’ ages ranged from 18 to 34 with an average age of 22.4 years, and the majority were enrolled in an educational institution (80%). We targeted this age group for two interrelated reasons. One, as emerging adults between the ages of 18 and 25 are navigating the transition from adolescence to adulthood, patterns of alcohol consumption and risktaking behaviors are prevalent and often increase (Anderson et al., 2020). Secondly, we included adults up to the age of 40 because time since traumatic or stigmatized event sometimes changes how they are seen or interpreted (Donde et al., 2018).

Participants were primarily female (60%) and White (87.5%). Other racial groups included Black (7.5%), Native Americans (7.5%), and Asian/Pacific Islander (5%). In terms of ethnicity, 7.5% of the sample indicated they were Hispanic/Latinx. Regarding sexual orientation, the majority identified as heterosexual, five (12.5%) identified as bisexual, one (2.5%) as gay/lesbian, and two (5%) as queer. The participants’ relationship status included 12 individuals identifying as single (30%), 19 individuals identifying as being in a monogamous relationship (19%), four individuals identifying as cohabitating or engaged with one partner (10%), three individuals identifying as married to one partner (7.5%), and one individual identifying as being in a polyamorous marriage (2.5%). Although participants were not asked to disclose, nine of them (22.5%) voluntarily disclosed that they had experienced sexual victimization.

Forty participants were recruited for this study by word of mouth, flyers, and the university’s SONA Research Pool. To be eligible for the study, participants must have been English-speaking and between the ages of 18–40. Informed consent was obtained prior to administering an online instrument that included the stimuli used in the interviews as well as a demographic questionnaire. We used a semi-structured interview protocol to encourage participants to compare and contrast items while allowing interviewers to ask clarifying follow-up questions. Interviewers were students supervised by the Principal Investigator. The cognitive interviews lasted on average 45 min. Upon the completion of this research study, participants received compensation for their participation with the option of receiving course credit or a $20 gift card.

Consistent with recommendations for cognitive interviewing (Beatty & Willis, 2007), interviewers used planned, proactive probes like, “How did you come up with your answer?” or “How did you figure out what it means? Walk me through your thinking as best you can.” Additionally, interviewers were trained to use reactive probes such as, “Tell me more about [unclear point].” Interviewers were instructed to read aloud the questions even as they appeared on the screen. Interviewers also used a timer to make sure they provided participants with sufficient time to formulate a response instead of jumping in with further prompts.

### Measures

Participants were presented with three statements and asked several questions about them. The statements described different scenarios of an AOD-involved rape scenario, and respondents were asked about their opinions of the different descriptions of rape. The statements presented to participants are shown in Table 1.

Questions asked to participants about these scenarios were:

1. What do these statements mean and how are they different?
2. What if someone had been roofied? Which item best fits their experience?
3. Does item C capture the things that are described in A and B?
4. Are there any other instances you can think of in which drugs or alcohol were involved that do not fit the descriptions in A, B, or C?

While items A–C reflect victimization, we designed prompts to examine comprehension and perceptions broadly, so that findings could be potentially applied to victimization or perpetration questionnaires. In addition to these directive prompts, interviewers were also able to ask follow-up questions to clarify any elicited responses.

### Data Analysis

Participants’ responses were recorded and transcribed in vivo by interviewers. After the interview, the transcripts of the interviews were reviewed for accuracy alongside the recordings by a research team member. The responses were de-identified and transferred to an Excel spreadsheet and coded and analyzed in Excel, using systematic manual coding (Ose, 2016). The research team qualitatively coded and analyzed the responses of 40 participants to the open-ended questions about the three different operationalizations of the AOD-involved rape question. The first three authors completed the initial coding procedures by developing themes, key words, and a comprehensive codebook including examples. Once the codebook was developed, the first three authors coded the same 12 case responses for inter-rater reliability checks and then met to discuss any discrepancies. The code definitions and examples were refined as a result and the coders continued with this process until they reached at least 80% agreement among the three of them (Kurasaki, 2000). Then, the third and fourth author coded the rest of the data according to the final codebook. The first, third, and fourth researchers met regularly and conducted regular quality control checks of the Excel data spreadsheet to enhance data accuracy and minimize errors. The research team then consolidated the codes into higher level, inter-connected themes that manifested most frequently in the data.

## Results

In response to questions about the differences between the three scenarios A, B, and C and their exhaustive description of AOD-involved rapes, respondents did not treat the scenarios as identical. Rather, they distinguished between levels of perpetration severity, as well as opportunistic versus intentional perpetration. Responses were consistent with respect to the question about being roofied. Lastly, respondents offered additional scenarios not covered by the three statements, as will be elaborated below.

Participants generally interpreted all three statements as describing scenarios of sexual victimization. As participant 234 summarized, “They mean if someone ever forced you to have any sexual relations without your consent, in this case it is involving alcohol or drugs.” Another participant commented: “These three statements are asking about has somebody ever performed unwanted sex with you in a state of intoxication” (participant 236). Another respondent commented on the commonality of the three scenarios: “I feel like most of these are all college party scene environments” (participant 133).

### Levels of Perpetration Severity

However, soon thereafter, most respondents distinguished between the three scenarios (A, B, and C) as describing different events of distinct severity of perpetration. Only one participant thought that “there really isn’t a difference between all of them because they all talk about substances and no consent” (participant 212), and five of them found only minor differences between the scenarios or felt that just one was different from the other two. Among the 85% who discussed the differences between the scenarios, one of them clearly stated,

They are all asking the same question that has someone ever taken advantage of you using drugs and alcohol. Each one of them are different situations [sic]. The first is asking has someone ever given [substances] to you and you didn’t know. B would be someone is trying to get you to do them and C is you’ve used some kind of substance and they decided to take advantage of you (participant 239).

Participant 222, for example, broadly described the three scenarios as covering varying levels of consent: “I think you covered the three levels of drugs and consent,” then elaborated on the perceived severity of each situation:

A is date rape to an extreme extent. They are actively drugging you, that is even worse than forcing. B still says the first part of the action is consensual. C is the less of the three but it is pretty clear they did not force you to have drugs or alcohol, but they still assaulted you.

Similarly, another participant viewed scenario A as the most severe degree of perpetration, B as moderate or less severe, and C as low levels of intentional perpetration:

I think it is about [the perpetrator’s] active role in getting you intoxicated. They are very different. [A] is someone is giving you drugs/alcohol without you knowing. [B] is you know you are drinking/drugs, but you’re pressured, [C] is you may or may not have done it without their encouragement, but they took advantage of the situation. A is much more premeditated than the other 2 (participant 229).

Another participant distinguished between different levels coercion by the perpetrator in the three scenarios:

I would say A is about date rape, drugs, or forcing alcohol in order to make it easier to sexually coerce you. B is about enabling or forcing, peer pressuring when it comes to drugs/alcohol with the intent to sexually assault or rape you. C is less about being forced to take alcohol/drugs and more about being taken advantage of after you were under the influence. You use alcohol or drugs on your own and they take advantage of you after the fact (participant 231).

Whether level of coercion or severity of violence was the distinguishing factor, the vast majority of participants viewed the three scenarios as distinct from one another. Respondents were able to pinpoint nuances in each scenario and to juxtapose the scenarios to one another. Other respondents emphasized the nature of perpetration.

### Opportunistic versus Intentional Perpetration

Another way several respondents distinguished between the three scenarios was by classifying the acts described in the three statements as either intentional or as taking advantage of the opportunity. For example, one respondent said,

The first one means that the person has been covertly given alcohol or drugs, they have been drugged so that they could take advantage of them. I think of a roofie. The second is I said I wasn’t drinking but someone is using peer pressure to get me to drink, and it becomes clear later they were doing that to pressure me into sex or not able to consent. The third is the implication is that I partook in drugs or alcohol on my own and I’ve gotten to the point where I have got to the place that I can’t consent, no one made me, but they are taking advantage of it (Participant 228).

Some respondents distinguished between the scenarios based on the intent they reflected. For example, one participant said, “The intent sounds different. A is has someone ever given this to you. B is encouraged or pressured you. C have you been taken advantage of” (Participant 237). Similarly, another respondent focused on the perpetrator’s intentions:

Yeah, I think that A is very intentional and without the individual’s knowledge. B is still very intentional, but the person is aware, they might just be feeling pressured or coerced into what’s happening. And the third one, they didn’t necessarily intentionally get you intoxicated, but they still violated you once you were (Participant 203).

Clearly, according to the participants, different intentions with respect to both intoxication and sexual violence underlie the three scenarios. A was perceived as the most intentional, then B, then C. It should be noted, however, that even the least intentional scenario was still perceived by respondents as describing a non-consensual sexual activity.

### Being Roofied

When participants were asked to identify phrasing that indicates someone had been roofied, they almost unanimously pointed to version A, “Has someone ever given you alcohol or drugs without your knowledge or consent in order to have sex with you without your consent.” Out of 40 respondents, 90% found this text to describe someone being roofied, regardless of sexual orientation. The few respondents who provided a detailed explanation for their choice seemed to focus on the consumption of substances: “It was without their knowledge, and it was not from being intoxicated so it is not C” (Participant 225). Another participant reiterated, “A, given drugs without consent or knowledge” (Participant 231). Another participant clarified, “A, if the person who took advantage of them is the one who gave it to them.” In other words, the identity of the sexual violence perpetrator as the one who also gave the victims the drugs was important for the classification of being roofied.

For the four participants who did not choose A, C seemed to be the common alternative. One participant chose C “because you were being taken advantage of” (Participant 214); two participants thought all scenarios could be described as being roofied (Participants 227, 204); and one participant selected A and B, “B because it has the word drugs. Actually A too. So A and B, just not C” (Participant 240). These participants relied on the word “drugs” as the relevant cue in the scenarios. Only one participant selected scenario B as being roofied, without providing explanation (Participant 236).

### Additional Scenarios and Judgments

While not directly related to the questions about the study items, participants made various judgment statements with respect to the three scenarios that went beyond their validity and pointed to the complexities of survey item interpretation. In their narratives, participants spoke about the challenges in assessing consent affected by AOD, the responsibility of the victim for getting intoxicated, and the need to specify both alcohol and drugs in the measures.

#### The Complexity of Measuring Consent Under the Influence of Alcohol

In response to whether the three scenarios captured all possibilities of AOD-involved rape, some respondents highlighted the difficulties of measuring consent under the influence of alcohol. A couple of participants focused on the coercive influence that alcohol may have, even at smaller consumption quantities:

There is definitely something to be said that even if you’re not necessarily too drunk to consent. I think that term is different based on the person. I think it is different based on the situation. Someone doesn’t have to be passed out to be too drunk to consent. A person could have a beer or 2 and it made it easier to continually pressure them into something that they would be more on guard for. I think that is very similar to B or C, but I think what I consider too intoxicated to give consent is not necessarily that I am not unable to be coerced. People can still recognize that (Participant 209).

In other words, this participant indicated that it is sufficient to be impaired by alcohol and not necessarily be fully drunk and incapacitated to be influenced heavily by alcohol and coerced to consent to things they would not do otherwise. Yet another participant emphasized the difficulty of judging the impact of alcohol by the quantity of consumption, saying, “It isn’t someone’s fault if they have one beer and all of the sudden pass out” (Participant 226). Another participant went further by talking about the possibility of willfully using substances when having sex without being fully intoxicated: “Yes, alcohol and drugs could be used in the lead up to sex, but [it] does not [necessarily] affect ability to consent” (Participant 221). Broadly speaking, then, according to these participants, the amount of alcohol the victim drinks or the victim’s level of intoxication are not necessarily good indications of the victim’s ability to consent to sexual activity.

Another respondent brought up a possibility that was not mentioned in any of the three scenarios, although it is common in real life: “If both partners were intoxicated, they’re intoxicated to the point where they can’t give consent, but they still have sex. That’s not covered within these questions, I guess” (Participant 236). Indeed, the three scenarios were silent on the level of intoxication of the perpetrator, which may or may not influence the victim’s perception of being raped and their subsequent reporting of the event.

#### Mentioning both Drugs and Alcohol

One respondent commented that statement C does not mention drugs, and if they were only reading that statement, they wouldn’t necessarily be thinking about passing out due to drugs:

Yes, except the drug part. If the person that is taking the survey is fully aware of all of these possible causes. I didn’t really think about being drugged, I would just think about being drunk, not drugged with some chemical (Participant 240).

Although only a single respondent made this comment, it is important to point out that indeed statement C does not explicitly mention drug use, so without the prior two statements, such phrasing could be misinterpreted by survey takers in a standalone item, as this participant indicated.

### Victim’s Responsibility for Substance Consumption

None of the items A–C mentioned substances willfully consumed by the victim. Although infrequent, some participants made comments that emphasized the victim’s responsibility in the scenarios. For example, one participant focused on the victim’s willingness to consume drugs or alcohol, saying,

A is like *you weren’t ever willing to consume alcohol or drugs*, and their sole purpose of giving it to you was to have sex; B was *you’re at least sort of willing to take those substances* and their sole purpose was still to have sex with you (Participant 205, emphasis added).

Yet another respondent spoke about the responsibility of the victim being “too intoxicated to consent” in the context of a sexual assault:

A is a little different, *B implies you played a role in the action*. You played some sort of role in this. The test taker isn’t assuming any responsibility. C the test taker doesn’t have any responsibility. *The only one which implies responsibility is B*. It could skew answers and create bias. It could also kind of mess with consent. Even though you were encouraged or pressured to do these things, *it still makes you feel somewhat responsible for it* (Participant 237, emphasis added).

Notably, participants were not asked nor probed about victim responsibility for anything in the three scenarios. Even if the responses may only highlight victim-blaming narratives within the survey language (and not the respondents’ opinions), their comments reflect the pervasive victim-blaming ideologies about the responsibility of the victim for potential consequences of excessive drinking and/or abusing drugs.

## Discussion

This paper, to the best of our knowledge, is the first to find evidence that items which measure AOD-involved sexual violence can be understood differentially by survey respondents based on item phrasing, supporting the construct validity of these items taken from the SES. Overall, participants demonstrated an understanding that all three scenarios involved sexual violence in the context of alcohol or drug use. We also identified themes representing some of the ideas participants used to distinguish the three scenarios: perceiving varying levels of severity in violence, intentions of the perpetrators, and the victim’s capability to consent. Although we did not ask specifically about victimization or perpetration experiences, and the items themselves alluded more specifically to victimization, respondents discussed both victimization and perpetration of sexual violence in their explanation of the items. The findings reveal important insights into the understanding of consent, responsibility, and victim responsibility in the context of AOD-involved rape. Thus, these items are a valid approach to measuring AOD-involved sexual violence and highlight the often-implicit perceptions participants hold that influence their interpretation of the items. The findings further support the application of multiple items in a single survey in order to capture the full gamut of AOD-involved sexual violence, rather than relying on a singular item.

### Distinction Between Items

Not only did respondents distinguish between the scenarios portrayed in items A, B, and C, but they also viewed them as representing different levels of severity. Scenario A, where someone gave drugs or alcohol without the victim’s knowledge or consent, was consistently described as the most severe. Scenario A was also most frequently identified as “being roofied.” The deliberate act of covertly drugging the victim was considered particularly egregious, surpassing even instances of forced sexual encounters. In contrast, scenario C, involving taking advantage of a victim who was already intoxicated or passed out, was perceived as less severe, though still constituting non-consensual sexual activity. Scenario B fell between the two in terms of severity, with participants considering the act of pressuring the victim to drink or take drugs until they were too intoxicated to consent as moderately severe. Some of the language used in these items resembles that on other sexual violence questionnaires (Abbey et al., 2005; Koss et al., 2007), and given the initial evidence of validity, it could be further tested and adapted into future questionnaires.

Another important distinction made by participants was related to the intentions of the perpetrators. Participants classified scenario A as the most deliberate, where the perpetrator actively gave drugs or alcohol to the victim with the purpose of facilitating non-consensual sexual acts. Scenario B, involving peer pressure to consume substances, was seen as intentional but less covert. Participants noted that in this scenario, the victim was aware of the substances being consumed but felt pressured or coerced into excessive intoxication. Scenario C, on the other hand, was perceived as less intentional, with the victim already being intoxicated and the perpetrator taking advantage of the opportunity. The perceived varying intentions of the perpetrators highlight participants’ recognition of different degrees of premeditation and coercion in the scenarios. These considerations could likely contribute to the classification of the scenario as a rape or not by survey respondents. Thus, even without explicit intention language in the item, participants assumed intent to rape on the part of the perpetrator, suggesting that questionnaire authors should focus on clearly describing behaviors and can ignore intent in survey items. That participants reacted to both perpetration and victimization perspectives within the items, even though items were copied from victimization questionnaires, suggests the language analyzed here has valid applications for both types of measurement.

Interestingly, although option C did not specify whether substance consumption was voluntary or not, respondents generally interpreted it as voluntary. In other words, the absence of a clear indication that the victim was intoxicated by someone else cleared the way for the assumption that the victim voluntarily brought themselves to an intoxicated state. This may be a manifestation of hostile attribution bias, whereby people tend to attribute negative behavior to others in ambiguous situations (Thomas & Weston, 2020). This finding indicates that survey items should be explicit in describing an AOD-involved rape scenario in terms of voluntary or involuntary AOD consumption.

In this context, it is noteworthy that in our study one participant expressed uncertainty about the presence of drugs and not just alcohol in Scenario C. This finding is similar to Jeffrey and Senn (2024) study, where one participant also expressed uncertainty whether intoxication items related to alcohol or to other substances as well. Questionnaire authors should keep in mind that if they want to measure both alcohol- and other drug-involved sexual violence, they should probably be explicit about both in their questionnaire item.

### Unexpected Findings

Apart from classifying the different scenarios, participants also raised some critical points for consideration, including contradicting judgments about victim’s responsibility for being intoxicated. Unexpectedly, because this was not included in our prompts, some participants expressed concerns about unjustified victim blaming, emphasizing that the amount of alcohol consumed should not solely determine a victim’s ability to give consent. These findings support Marcantonio and Jozkowski’s (2021) study, whose college participants also expressed confidence in their consent to sexual activity at lower levels of drinking-related impairment. Survey language should be careful not to encourage judgment and inadvertent victim blaming, which is important for both victimized respondents and perpetrator respondents.

Research has indicated that alcohol, as a construct related to sexual assault, is complicated (Chiu et al., under review; Muehlenhard et al., 2016). Our findings are no different. When parties are drunk and have to negotiate consent, this process may result in confusion and misunderstandings (Ward et al., 2012). It is possible that such confusion extends to the reporting of such ambiguous situations in surveys; research suggests that when participants are uncertain, they will underreport their experiences of violence (Evans et al., 2016). Clearly, per our respondents, drinking does not automatically equate to non-consent or inability to consent to sexual activity. Unlike Ullman et al.’s (2019) study, some of our respondents distinguished between consenting under a state of being only impaired by alcohol and being fully incapacitated by it. Thus, questionnaire authors should focus on different ways to express consent and describe the varying impacts of AOD within items.

In contrast, it is important to acknowledge that a small number of participants made comments that did reflect victim-blaming ideologies. These participants specifically pointed to the responsibility of the victims for their alcohol consumption in some of the scenarios, suggesting that the victim’s willingness to consume drugs or alcohol and their level of intoxication (i.e., if they reached a stage of incapacitation—“passed out”) were factors that should be considered in the context of sexual violence. In other words, if some respondents believe that the victim’s intoxication was self-imposed, they may doubt their status and legitimacy as a victim of sexual violence and consequently underreport. Such an interpretation is congruent with Orchowski and colleagues’ (2022) study, which found that in drinking environments, men make assumptions about women being interested in sexual activity if they drink with them; these assumptions result in expectations which may be at least partially responsible for sexual violence in drinking environments, although the men do not perceive their acts as violent. Some prior research has suggested that when assaults are consistent with victim-blaming stereotypes, they may be underreported (Anderson et al., 2020). Thus, including explicitly victim-empowering statements, or carefully describing the drinking situation, may be strategies to improve recall and therefore accuracy in measuring AOD sexual violence.

Additionally, respondents with prior victimization histories may feel offended when responding to an item that contains implicit victim-responsibility language (Houston-Kolink & Vasquez, 2022). This suggests that researchers may benefit from using instruction sets which minimize blame and apply behaviorally specific language that may subvert stereotypes, for example, indicating that the victim was not willingly intoxicated. As McMahon and Farmer (2011) demonstrated in the context subtle sexism and rape myth beliefs, sometimes subtle changes in wording or setting description have a large impact on responses in the context of sexual assault.

### Importance of Context

Respondents clearly took account of the responsibility for creating the situation in which sexual violence was facilitated, and the implicit reasons for drinking and/or using drugs. Participants generally viewed opportunistic rape, where the victim was already incapacitated, as less severe. They also made assumptions in cases where information was missing. For example, in scenario C, where the victim was already intoxicated but no information was provided about the causes of intoxication, respondents assumed that the intoxication was self-imposed. In other words, respondents “filled in the blanks” with imaginary scenarios in order to respond to the item. This is in line with findings from other cognitive interview studies. For example, participants in Mason et al.’s (2023) cognitive interviews grappled with the lack of nuance that questionnaire measures displayed. According to their study participants, the quantitative measures did not fully capture the rich nuance of participants’ responses, particularly if these participants belonged to marginalized groups. Such nuance should also be added to items which measure AOD-involved sexual violence; at the very least, it should be clarified who is responsible for the victim’s intoxication.

Nuance typically requires context. Researchers have traditionally used case vignettes in studies designed to measure sexual consent (Ward et al., 2012). Based on the present study’s results, it may be appropriate to apply a revised version of this methodology to AOD-involved sexual violence items to reduce question ambiguity. For example, in place of scenario C as currently phrased, respondents could be asked:

Think of a time when you were at a party where alcohol was consumed. You were with a group that was constantly providing you and others with drinks, until you passed out on a couch in a side room. Has anyone in such a situation ever taken advantage of you when you were unable to consent or stop what was happening in order to have sex with you?

Of course, researchers should be mindful that once context is used in a question, respondents may ignore other contexts in which sexual violence may have happened to them. Future research should assess response rates to questions with and without vignettes, to determine which type of question elicits more exhaustive responses from participants.

The findings demonstrate that at least when asked about AOD-involved rape, behavioral context is important and can make a difference in the measurement of sexual violence. As Graham et al. (2023) have demonstrated, using short vignettes to describe the drinking venue and the specific behavior perpetrated yielded more responses of sexual aggression perpetration compared to traditional surveys such as the SES that only list the behavior without context. For instance, respondents in their study were instructed as follows:

Think about times when you were at a bar or club in the 6 months prior to the COVID-19 pandemic. How often have you done anything like what the guy(s) did in each of the following (e.g., A guy keeps trying to kiss a girl he doesn’t know after she has pushed him away)? In all descriptions, both the guy(s) and the girl in their early 20s are at a bar or club and they do not know each other (Graham et al., 2023, pp. 4–5).

While Graham et al.’s study did not test more severe form of sexual aggression in drinking settings such as rape or attempted rape, there is no reason to believe that providing context would improve measurement of incidents for severe forms of sexual violence as well.

### Being Roofied

In contrast with the ambiguity of scenario C, the majority of participants identified scenario A as indicative of someone being “roofied,” where drugs or alcohol were given to the victim without their knowledge or consent in order to facilitate a sexual assault. If a survey aims to ask about experiences of sexual assault after being drugged, our findings indicate that the best phrasing is “Has someone ever given you alcohol or drugs without your knowledge or consent in order to have sex with you without your consent?”. Our respondents relied primarily on the victim receiving substances without awareness. However, a small subset of participants also considered scenario C as a possible indication of being roofied, highlighting the role of interpretation and individual understanding. Based on other responses, it is important to reference both alcohol and drugs explicitly in the description of the scenario.

Although not as common, some college-age respondents may find all three scenarios to depict the commonality of being “roofie” scenario, as was expressed by one of the participants who viewed all three scenarios as “college party scene environments.” In other words, according to this one participant, AOD-involved sexual violence is a normal part of college culture. To them, all three scenarios allude to sexual violence on college campuses. While the participant’s comment about the commonality of such scenes in college parties may be striking, it is in no way surprising. Rather, it reflects and supports the findings from many studies about the prevalence of rape among one in five women and one in 16 men in college settings (Armstrong et al., 2006; Lim & Roloff, 1999; Ward et al., 2012). Indeed, due to the commonality of these scenarios, Graham et al. (2023) have pointed to the need for a series of standardized items to measure sexual aggression that are common in drinking settings, as opposed to measuring it ad hoc, often with a single item. Our findings, which offer diverse interpretations of a single item, support this recommendation.

### Implications

The findings of this study support the validity of the tested items. Our findings also point to specific considerations in the wording and phrasing of survey items for both victimization and perpetration questionnaires, since ambiguity (such as in Scenario C) can inadvertently contribute to participants’ responses and introduce response biases. Alcohol and drugs should both be mentioned in the item, as well as who consumed them, whether consumption was voluntary or not, and who initiated or was responsible for the drinking. Survey language should strive to avoid instances of potential victim blaming.

The present study also highlights the potential value of incorporating contextual information in survey scenarios. Real-life situations involving AOD-involved rape are complex and multifaceted, influenced by various situational factors, relationships, and individual characteristics. Our findings indicate that such complexities have a tangible contribution to participants’ interpretations of survey items. Similar to the measure developed by Graham et al., we recommend using a vignette with a single-item AOD-involved rape question, in order to provide respondents with more context and nuance with which to interpret the survey item. The use of vignettes could not only provide additional useful information, but also minimize potential victim blaming and clarify the intentions of those involved in the scenario. At the very least, future surveys should aim to provide a more realistic and nuanced portrayal of scenarios by including contextual details, such as relationship dynamics, intoxication levels of both/all parties, and relevant situational cues. This may help researchers better capture the complexities of AOD-involved sexual victimization and consent within specific situations.

While the current paper focused on improving the measurement of AOD-involved sexual violence, the narratives of our participants also have some important implications for prevention and intervention programs. No sexual and dating violence prevention programs examined in Verbeek et al.’s (2023) systematic review discussed alcohol in the context of consent (Verbeek et al., 2023). Our study findings demonstrate the need to address covert drugging, verbal coercion to drink, and taking advantage of intoxicated individuals. Moreover, such prevention interventions should discuss victim blaming in AOD-involved sexual violence scenarios and convey the notion of perpetrator responsibility regardless of levels of intentionality or intoxication. The focus should be on the impairing role of AOD on consent, even if the victim chooses to drink and/or use drugs, rather than on the decision to drink/use drugs itself. Emphasizing the importance of consent and responsibility in the context of AOD may help reduce substance-involved rape (Katz et al., 2015).

### Limitations

Although our sample was mostly young in age, majority White, and primarily cisgender women, all of the themes identified were mentioned by men and women and by those with dominant (i.e., White) and minoritized racial identities. While young, our sample was drawn mostly from a college-aged population and a national community pool that is the most likely to experience sexual assault and be surveyed about it (for example, in university psychology department pools). Notably, nine participants volunteered that they had experienced sexual violence and this may have informed their responses. Therefore, it is the appropriate population to survey about sexual violence-based survey items.

Another limitation concerns the fact that some participants struggled to respond to the specific items for various reasons. Perhaps including more probes about emotional reactions would have helped them further articulate their thoughts. Specifically, for this research, we only used the cognitive interviewing protocol. Future studies can accompany this protocol by the think-aloud protocol, whereby participants are encouraged to constantly verbalize their thoughts about and not just their understanding of the items (for the differences between these two protocols, see Wolcott & Lobczowski, 2021). Indeed, Mason et al. (2023) recommend using think-aloud protocol alongside cognitive interviews to improve data quality in psychological research.

Finally, while 20% of the sample (*n* = 8) identified as a sexual minority, this sub-sample was too small to distinguish similarities and differences in responses based on sexual orientation across different populations. Future research could address this point and recruit comparable sub-samples of different sexual orientations. Doing so would improve our understanding of how sexual orientation might influence responses, which could inform more tailored policies and interventions for targeted populations.

### Future Research

The scenarios presented to participants in this study were limited in their contextual information. By excluding behavioral context about the scenarios, some important factors that could affect how participants see the situations might have been overlooked or not fully understood. Future studies should assess the value of using vignettes before asking respondents to reflect on particular phrasing of questions related to the scenarios in those vignettes as suggested above. While doing so, they should also determine whether particular vignettes may exclude other contexts, for instance, by asking respondents as the current study did, “Are there other scenarios that are not covered in the vignette?” It would also be interesting to replicate the study while contextualizing it in different legal landscapes, in order to see whether the legal definition of rape or sexual assault, which varies across states, has any impact on respondents’ perceptions of AOD-involved sexual violence.

Considering our respondents’ perspectives related to substances and consensual sex, it would be interesting to explore in future research other motivations to use alcohol during sex, and whether they are perceived as legitimate by respondents. For example, as some participants noted, the three scenarios did not explicitly address the level of intoxication of the perpetrator, or whether additional motivations for alcohol during sex, such as enhancement of pleasant social interactions (Foster & Neighbors, 2013) or tension reduction motivations (Corbin et al., 2001), can make up for lack of clear consent. The results could shed light on the obfuscating role of alcohol during sexual assault, and such improved understanding could in turn contribute to better phrasing of alcohol-involved rape scenario survey items and improve the measurement of AOD-involved sexual violence.

## Conclusion

Our findings demonstrate first and foremost that language matters: Different phrasings of an AOD-involved rape scenario elicit different interpretations among readers. Although respondents identified all three scenarios as describing sexual violence, they pointed to important nuances in each scenario: recognizing varying levels of severity, intentions of perpetrators, and the implications for victim consent. As Mason and colleagues (2023) have previously demonstrated, this study has also shown that there is more to filling out questionnaires than what is initially apparent to academics. The nuances identified by the respondents point to the need to incorporate multiple items in a questionnaire which measures substance-involved sexual violence, in order to more effectively capture incidents of AOD-involved sexual violence. If scholars want to build a richer, more participant-informed validation of measurement items, they should integrate qualitative perspectives (Mason et al., 2023), enhancing the validity and relevance of the measurement tools.

## Figures and Tables

**Table 1:** Alcohol and other drugs-involved sexual violence survey item descriptions

Questions	Type of consumption	Degree of impact	Sources
Has someone ever given you alcohol or drugs without your knowledge or consent in order to have sex with you without your consent?	Completely involuntary	Unclear	Abbey et al. (2005)
Has someone ever encouraged or pressured you to drink alcohol or take drugs until you were too intoxicated to consent or stop what was happening in order to have sex with you without your consent?	At least somewhat involuntary	Incapacitated	Koss et al. (2007)
Has someone ever taken advantage of you when you were passed out or too intoxicated to give consent or stop what was happening in order to have sex with you without your consent?	Unclear	Incapacitated	Abbey et al. (2005)

## Data Availability

Data from this study are not publicly available and may be requested from the last author.

## Footnotes

1 The term “being roofied” typically refers to being drugged without consent and is often associated with date rape drugs like GHB or Rohypnol, one of whose street names is “roofie” (Negrusz & Gaensslen, 2003). Often referred to as “drink spiking,” the common scenario occurs when a hypnotic, sedative, or anxiolytic substance is covertly added to an alcoholic beverage, allowing the offender to carry out their sexual assault (Benyon et al., 2008). Rohypnol and GHB are clear and odorless, dissolve quickly in liquid and cause loss of consciousness and memory loss. They also disappear from the victim’s blood within 72 h, making it difficult to measure the frequency of being roofied (Blendermann, 2023).
